# The SIDER database of drugs and side effects

**DOI:** 10.1093/nar/gkv1075

**Published:** 2015-10-19

**Authors:** Michael Kuhn, Ivica Letunic, Lars Juhl Jensen, Peer Bork

**Affiliations:** 1 Max Planck Institute of Molecular Cell Biology and Genetics, Pfotenhauerstr. 108, 01307 Dresden, Germany; 2 Biobyte solutions GmbH, Bothestr. 142, 69117 Heidelberg, Germany; 3 Novo Nordisk Foundation Center for Protein Research, Faculty of Health and Medical Sciences, University of Copenhagen, 2200 Copenhagen N, Denmark; 4 European Molecular Biology Laboratory, Structural and Computational Biology Unit, Molecular Medicine Partnership Unit, Meyerhofstrasse 1, 69117 Heidelberg, Germany; 5 Max-Delbrück-Centre for Molecular Medicine, Robert-Rössle-Strasse 10, 13092 Berlin, Germany

## Abstract

Unwanted side effects of drugs are a burden on patients and a severe impediment in the development of new drugs. At the same time, adverse drug reactions (ADRs) recorded during clinical trials are an important source of human phenotypic data. It is therefore essential to combine data on drugs, targets and side effects into a more complete picture of the therapeutic mechanism of actions of drugs and the ways in which they cause adverse reactions. To this end, we have created the SIDER (‘Side Effect Resource’, http://sideeffects.embl.de) database of drugs and ADRs. The current release, SIDER 4, contains data on 1430 drugs, 5880 ADRs and 140 064 drug–ADR pairs, which is an increase of 40% compared to the previous version. For more fine-grained analyses, we extracted the frequency with which side effects occur from the package inserts. This information is available for 39% of drug–ADR pairs, 19% of which can be compared to the frequency under placebo treatment. SIDER furthermore contains a data set of drug indications, extracted from the package inserts using Natural Language Processing. These drug indications are used to reduce the rate of false positives by identifying medical terms that do not correspond to ADRs.

## INTRODUCTION

Reducing adverse drugs reactions (ADRs) and elucidating their origin has long been a concern of physicians and researchers within their respective fields of medicine. Within the last years, an increased availability of public data on drug targets and ADRs enabled large-scale studies that go beyond individual fields of medicine to a systems biology or systems medicine approach. For example, it was shown that ADRs can be used to predict drug targets (1) and that causal relations between targets and ADRs can be elucidated (2,3).

Data on ADRs are generated in two stages. First, during placebo-controlled clinical trials, the occurrence and frequency of ADRs is recorded. In phase III trials, thousands of patients are carefully monitored, and the ADRs observed during this phase become listed on the package inserts. Once the drug is on the market, surveillance continues (‘phase IV’) and doctors may report ADRs to systems such as the FDA Adverse Event Reporting System (AERS). ADRs from such reporting systems are drawn from much larger numbers of patients; however, they are more subject to confounding biases and the causality may thus be questionable. Typically, such ADRs are also added to package inserts in a separate section on post-marketing experience. In addition, ADRs are reported in the biomedical literature, e.g. in animal studies or in pre- or post-clinical trial studies, and in electronic health records, from where they can be extracted using text mining (4–6).

To make ADRs amendable to academic research in a simple way, we developed SIDER (‘Side Effect Resource’) in 2010 when no such resource was freely available for academic researchers (7). The first version of SIDER contained not only drugs and their respective ADRs but also frequency information for both drug and placebo treatment. Users were able to check for the occurrence of specific ADR on the SIDER website. Thanks to the availability of downloadable files, SIDER has since been used in many studies, for example to identify metabolic dysregulation as a cause for ADRs (8), to investigate the effect of essential proteins on ADRs (9) and to predict drug indications (10). As a further use case, SIDER has been used as the benchmarking set for text-mining methods that extract ADR data from the literature (4,11), and other databases have incorporated data from SIDER. For example, ADReCS combined SIDER 2 data with an independent annotation effort and further added an ontology of ADRs that allows grouping of related ADRs (12). IntSide integrated data from SIDER 2 with drug–target and pathway data to pinpoint causes of ADRs (13). Despite no longer being the only freely available ADR resource, SIDER remains heavily used: on average, the SIDER 2 data set of ADRs was downloaded 74 times per month in the past year (unique IP addresses per month, August 2014 to July 2015).

We present here a new release of the SIDER database with over 40% more drugs, ADRs and drug–ADR pairs compared to the previous version and more than 2-fold as many drug–ADR pairs as the published version (see Table 1). We ensure a high quality of the extracted entities by manually annotating names, adding synonymous names and using an additional Natural Language Processing step. Our text-mining system creates a machine-readable database and a human-readable website with highlighted terms at the same time, making it possible for users to quickly trace the origin of extracted ADRs.

**Table 1.** Data content of ADR databases

	ADReCS 1.2 *	SIDER 1	SIDER 2	SIDER 4	Increase between SIDER 2 and 4
Year of release	2015	2009	2012	2015	
Number of drugs	1378	888	996	1430	+44%
Number of ADRs	5984	1450	4192	5880	+40%
**Number of drug–ADR pairs**					
total	134022	62269	99423	140064	+41%
with frequency	50490	25068	42331	54772	+29%
with frequency for placebo	0	3640	6334	10790	+70%

*ADReCS statistics were accessed on July 30, 2015.

## DATA COLLECTION AND QUALITY CONTROL

Drug labels with information for professionals were obtained from national registries and charity organizations. Structured Product Labels, as provided by the FDA in XML format, could be parsed directly. Labels that were only available as PDF were converted to HTML, preserving tabular data formatting to the extent possible. The initial conversion from the source documents to PDF removes the logical structure of the documents such as headings. Converting from PDF to HTML yields documents with different styles for normal text and headings, but the actual text formatting varies between documents. We therefore developed heuristics to automatically identify section and subsection headings from the text formatting in the HTML labels. For example, we search for headings of the ADR and indications section with different wordings to identify the text style used to indicate the different sections. We also maintain a list of section titles that indicate that the ADR or indication section have ended (e.g. ‘Interactions with other drugs’).

### Named entity recognition (NER)

We used a dictionary-based approach to detect mentions of ADRs, diseases, drugs and proteins in the package inserts. Names of chemicals and proteins were taken from the STITCH 4 and STRING 9.1 databases (14,15). In particular, users can directly use identifiers from SIDER to identify drug targets in STITCH 4. In STITCH, stereoisomers and salt forms are usually merged into a common identifier, although the user has the option to view the isomers separately. Likewise, the SIDER download files contain identifiers both with and without stereochemistry, and links to PubChem are provided on the web frontend. Starting with SIDER 4, the major version number of SIDER is linked to the corresponding STITCH version. That is, once STITCH 5 has been released, we will create SIDER 5 with new chemical identifiers. To create a dictionary of ADR and disease names, we pooled synonyms from the Unified Medical Language System (UMLS) Metathesaurus (version 2014AA) for all terms of the Medical Dictionary for Regulatory Activities (MedDRA) (version 16.1). We filtered by semantic type (Supplementary Table S1) and further manually created an exclusion list of concepts with the correct semantic type that did not refer to ADR, such as ‘HIV positive’ or ‘family history of suicide.’ Similarly, we manually inspected all names that occurred at least 100 times during NER to create a list of names to be blocked from the dictionary.

Names from the UMLS Metathesaurus often do not cover all possible permutations of words or their synonyms. To expand the dictionary, we created a list of candidate synonyms from frequently interchanged words. For each concept, we pooled the words of all names and added synonymous words. Then we scanned the package inserts for occurrences of these words. In this way, mentions of the concept could be detected even when the order of words had been changed. For example, consider the name ‘blood pressure decreased’. After adding a synonym, the algorithm might look for the words ‘blood’, ‘pressure’, ‘decreased’ and ‘lower’. It would therefore find new names like ‘decreased blood pressure’ and ‘lower blood pressure’. We manually annotated the most frequently occurring novel names and added only truly synonymous names to the dictionary. After completing the dictionaries, entities were recognized using an NER engine that also accounts for orthographic variation, specifically case variation and insertion/removal of hyphens and spaces (16). Sentences in the ADR section that contain negations (e.g. ‘has not been observed’) or speculation (e.g. ‘is suspected’) were excluded (see Supplementary Table S2).

**Figure 1.**
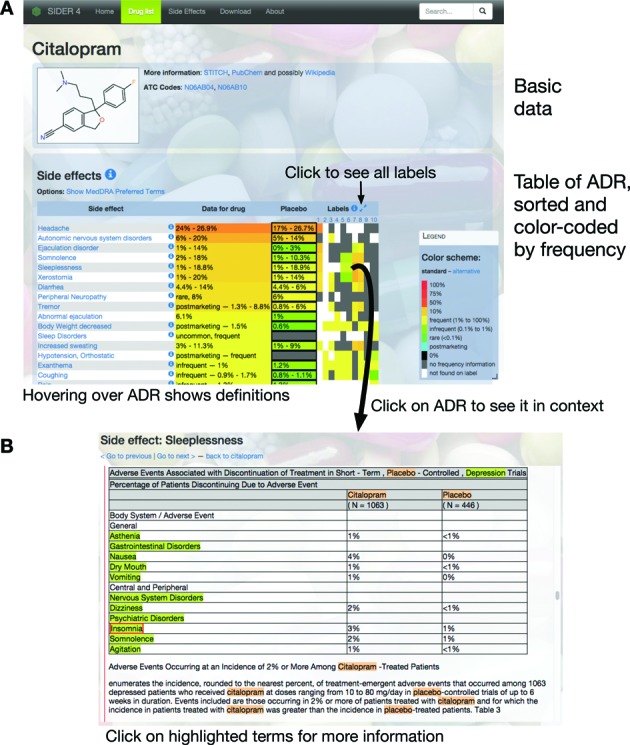
Navigating the SIDER website. Users can search for drugs to get an overview of its ADR (**A**). Clicking on cells in the table of labels and ADRs takes the user to a separate page (**B**) where they can inspect all mentions of the ADR.

By tightly integrating parsing of the labels and entity detection, we were able to produce marked-up HTML versions of the labels. In these files, all detected mentions of the ADR terms are highlighted, along with names of chemical compounds and proteins (Figure 1). In previous versions of SIDER, highlighting of matches and entity recognition were independent steps, which meant that in some cases the source of a detected term was not highlighted in the HTML version. Users can click on ADR terms to get more information from SIDER, and on proteins and compounds for relevant data from Reflect (17).

### Detection of drug indications by natural language processing

After entity recognition, we used the Stanford Dependencies (18) package (version 3.4.1) to extract further information from the package inserts, analyzing the content of the sections on indications and ADRs. This Natural Language Processing (NLP) approach makes it possible to identify sentence fragments that refer to indications of drugs. As a pre-processing step, we replaced each entity that had been recognized in the previous step with an internal identifier. This way, entity names that consisted of multiple words were collapsed to a single noun, making correct parsing of the sentences easier. With the preprocessed sentences as input, Stanford Dependencies returns a list of dependencies, which are triplets consisting of the kind of dependency and the two connected words (the governor and the dependent). For example, parsing the sentence ‘DRUG may be used for the treatment of INDICATION’ returns this list of dependencies, consisting of seven triplets:

nsubjpass(used-4, DRUG-1)
aux(used-4, may-2)
auxpass(used-4, be-3)
root(ROOT-0, used-4)
det(treatment-7, the-6)
prep_for(used-4, treatment-7)
prep_of(treatment-7, INDICATION-9)

Analyzing this list of dependencies, we extracted the triplet ‘DRUG, used treatment, INDICATION’ and used custom rules to determine that this implies an indication for the drug (Supplementary Table S3). We used such rules to detect drug combinations, indications and pre-existing conditions of patients that do not refer to ADRs (e.g. ‘In patients with X, Y may occur’, see Supplementary Table S4).

### Extraction of frequencies

Frequencies of ADRs were extracted from tables and free-text mentions. In case of tables, the header of the table was analyzed to find out if the reported frequencies are for drug or placebo treatment. We used a heuristic to exclude tables that contain other types of data, such as discontinuation frequencies. We also extracted frequency information from the text of the labels, searching for lists (e.g. ‘frequent: headache, fatigue’) and for numbers in parentheses (e.g. ‘headache (12%)’).

### Filtering steps

To further reduce the rate of false positives, the following steps were undertaken. First, we manually inspected the words found at least 100 times and removed terms that did not correspond to ADRs or were ambiguous. Next, we used the extracted indications as a filter on ADR found in the free text of the ADR section (in contrast to those contained in tables listing the frequency of ADR). As described above, potential indications of drugs were detected in two ways: either by NER in the indications section, or by NLP (yielding indications and pre-existing conditions of patients). When a concept had been found by NER in the ADR section of a package insert, we discarded it as an ADR if the concept had been detected (i) by NLP on any package insert of the same drug or (ii) by NER in the indications section of the same package insert. In the latter case, filtering only applies to the same package insert due to a higher rate of false positive indications for NER compared to NLP shown below.

To estimate the accuracy of the extracted indications, we matched them against a data set of drug indications derived from the resource drugs.com. We looked for exactly matching terms and used this external data set only for benchmarking the final set of indications (see Table 2). Among the overlapping drug–disease pairs, 61% of those found as an indication by NLP were also contained in the drugs.com data set. For those detected as preconditions, the fraction was 53%. Among terms found by text mining in the indications section, 44% were also contained in the external data set.

**Table 2.** Comparison of extracted indications and ADRs between SIDER 4 and drugs.com (accessed on February 12, 2015)

	Indications (detected by NLP)	Pre-existing conditions (detected by NLP)	Indications (detected by NER)	ADRs (filtered set)
**Counts within SIDER 4**				
drugs	1236	826	1329	1430
concepts	1338	809	2583	880
drug–concept pairs	4681	2616	12965	140064
**Comparison with** **drugs.com**				
common drugs	870	615	915	948
common concepts	439	292	553	480
**Number of drug–concept pairs**				
only in drugs.com	1328	1020	1034	2216
intersection	1010	499	1685	349*
only in SIDER 4	655	434	2111	27797
**Overlap**				
relative to SIDER 4	61%	53%	44%	1.2%
relative to drugs.com	43%	33%	62%	13%

*This intersection should be as low as possible, as it hints at false positives in SIDER, see discussion in text.

We also used the drug indication data from drugs.com to obtain an estimate of the false positive rate of ADR in SIDER. In the previous paragraph, we quantified the overlap between indications in drugs.com and SIDER. Here, we tested the overlap between indications from drugs.com and ADR found in SIDER. Thus, matching terms point to an apparent contradiction that could be explained as a false positive in either drugs.com or SIDER. Of 140064 drug–ADR pairs in SIDER, 28343 (20%) could be matched to drugs and diseases found on drugs.com. Of those 28343 drug–disease pairs, only 349 (1.2%) were listed as drug–indication pairs on drugs.com, showing that indications are only very rarely misinterpreted as ADRs.

## DATA CONTENT AND AVAILABILITY

**Figure 2.**
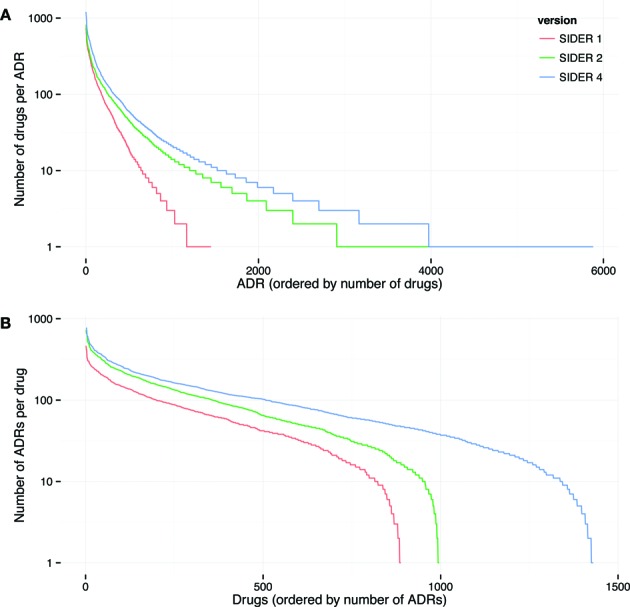
Data content of SIDER. For SIDER versions 1, 2 and 4 the distribution of (**A**) drugs per ADR and (**B**) ADRs per drug is shown. These distributions do not follow a power law. Note that the current version of SIDER is the third release, but is designated SIDER 4 because it is based on the same set of chemicals as STITCH 4.

SIDER is available at http://sideeffects.embl.de/. The new release, SIDER 4, represents a large increase in the numbers of drugs, ADRs, drug–ADR pairs and drug frequency entries compared to previous versions (Table 1, Figure 2). The homepage allows users to interactively browse the database and to search for drugs and ADRs (Figure 1A). The SIDER website enables users to trace drug–side effect pairs to the drug labels: users can navigate to the drug's page and click on the side effect of interest. On the presented drug label, all instances of the side effect are marked (Figure 1B). In this way, users can quickly trace the origin of an extracted side effect in order to rule out false positives. The complete data set of side effects and the data set of indications are available for download from the SIDER website in text format, including PubChem and MedDRA identifiers. In addition, we have created a GitHub repository (linked from the SIDER download page) where users can contribute errors that they detect in SIDER. In this way, other users can filter the download files, and the authors can remove the source of the errors in upcoming versions of SIDER.
